# Health-related quality of life in long-term survivors of colorectal cancer and its association with all-cause mortality: a German cohort study

**DOI:** 10.1186/s12885-018-5075-1

**Published:** 2018-11-22

**Authors:** Ilka Ratjen, Clemens Schafmayer, Janna Enderle, Romina di Giuseppe, Sabina Waniek, Manja Koch, Greta Burmeister, Ute Nöthlings, Jochen Hampe, Sabrina Schlesinger, Wolfgang Lieb

**Affiliations:** 1 Institute of Epidemiology, University of Kiel, University Hospital Schleswig-Holstein, Niemannsweg 11, 24105 Kiel, Germany; 2 Department of General and Thoracic Surgery, University Hospital Schleswig-Holstein, Kiel, Germany; 3 Department of Nutrition, Harvard T.H. Chan School of Public Health, Boston, MA USA; 4 Nutritional Epidemiology, Department of Nutrition and Food Science, Rheinische Friedrich-Wilhelms-University Bonn, Bonn, Germany; 5 Medical Department 1, University Hospital Dresden, Technical University Dresden, Dresden, Germany; 6 Institute of Biometrics and Epidemiology, German Diabetes Center at Heinrich Heine University, Leibniz Institute for Diabetes Research, Düsseldorf, Germany

**Keywords:** Health-related quality of life, Long-term survivors, Colorectal cancer, Correlates, Mortality

## Abstract

**Background:**

The group of colorectal cancer (CRC) survivors continues to grow worldwide. Understanding health-related quality of life (HRQOL) determinants and consequences of HRQOL impairments in long-term CRC survivors may help to individualize survivorship care plans. We aimed to i) examine the HRQOL status of CRC long-term survivors, ii) identify cross-sectional sociodemographic and clinical correlates of HRQOL, and iii) investigate the prospective association of HRQOL after CRC diagnosis with all-cause mortality.

**Methods:**

We assessed HRQOL within a Northern German cohort of 1294 CRC survivors at a median of 6 years after CRC diagnosis using the European Organisation for Research and Treatment of Cancer Quality of Life Questionnaire Core 30 (EORTC QLQ-C30). Cross-sectional correlates of different HRQOL dimensions were analyzed using multivariable-adjusted logistic regression models with HRQOL as a binary variable. With multivariable-adjusted Cox proportional hazards regression models, hazard ratios (HR) of all-cause mortality were estimated per 10-point-increments of an HRQOL summary score, a global quality of life scale, and HRQOL functioning and symptom domains.

**Results:**

The median HRQOL summary score was 87 (interquartile range: 75–94). Sex, age, education, tumor location, metastases, other cancers, type of therapy, and current stoma were identified as correlates of different HRQOL scales. After a median follow-up time of 7 years after HRQOL assessment, 175 participants had died. Nearly all HRQOL domains, except for cognitive functioning and diarrhea, were significantly associated with all-cause mortality. A 10-point-increment in the summary score decreased the risk of death by 24% (HR: 0.76; 95% CI: 0.70–0.82).

**Conclusions:**

HRQOL in CRC survivors appeared to be relatively high in the long term. Various clinical and sociodemographic factors were cross-sectionally associated with HRQOL in long-term CRC survivors. Lower HRQOL was associated with increased all-cause mortality. Individualized healthcare programs for CRC survivors (including psychosocial screening and interventions) are needed to detect decreased HRQOL and to further improve long-term HRQOL and survival.

**Electronic supplementary material:**

The online version of this article (10.1186/s12885-018-5075-1) contains supplementary material, which is available to authorized users.

## Background

As the group of patients surviving colorectal cancer (CRC) is growing, understanding and improving health-related quality of life (HRQOL) in these patients is becoming an important field of research [1, 2]. CRC survivors may be impaired in physical functioning and in everyday life by multiple disease- and treatment-related symptoms such as pain, bowel dysfunction, and fatigue and may be negatively affected in psychological, emotional, social, and role functioning because of fear, anxiety, sleep disruption, and depression [3–6]. Therefore, assessing and - if necessary - improving physical, social, psychological, and sexual function and well-being in CRC survivors is pivotal [7]. Assessment of HRQOL in CRC survivors provides insight into their experiences of the disease, therapy, and recovery, helps to identify risk factors of low HRQOL, and might support the choice and design of appropriate interventions and survivorship care plans [8–10].

HRQOL in CRC survivors has been addressed in prior studies, but most of these studies evaluated rather short-term (≤5 years after diagnosis) treatment- and disease-related effects on quality of life (QOL) [11–14]. A few studies investigated HRQOL in patients who survived at least 5 years after CRC diagnosis but most of them relied on relatively small sample sizes [1, 15–18]. In two studies in the US, a relatively high QOL was observed in 227 and 173 CRC survivors, respectively, with QOL obtained ≥5 years after CRC diagnosis [1, 15]. A recent study with 296 CRC survivors obtained HRQOL on average 15 years after the cancer diagnosis and reported comparable or even slightly higher quality of life in cancer survivors than in age- and sex-matched unaffected controls [18]. Similarly, in a cohort of 6952 long-term cancer survivors, overall HRQOL was comparable to 1878 population-based controls, but differences with respect to specific functioning and symptom scales were reported [19]. Furthermore, higher prevalence of depression and anxiety in CRC survivors as compared to the general population have been reported in several studies [1, 10, 20]. With respect to factors influencing QOL, different clinical, sociodemographic, and lifestyle factors, including age, sex, tumor location, body mass index (BMI), stoma, and physical activity, were associated with HRQOL of CRC survivors in previous epidemiological studies [8, 21–23], even though findings were partially inconsistent in terms of their effect sizes and effect directions. In a previous investigation, we have examined the relation between selected lifestyle factors (diet, BMI, physical activity, and smoking status), modeled as a lifestyle index, and HRQOL in our CRC survivor cohort [24] and observed that a favorable diet, more physical activity, and lower BMI were significantly associated with higher HRQOL. In the present study, we will expand on this previous analysis by i) investigating the association of a broad panel of clinical and sociodemographic factors (not considered in our prior analyses [24]) with HRQOL and ii) relating HRQOL prospectively to all-cause mortality. To our knowledge, so far, only one study examined the association between HRQOL and mortality in long-term CRC survivors and provided initial evidence for an inverse relation between physical and mental component scores and mortality risk [22].

Thus, the aim of this study was three-fold: first, to describe the HRQOL status of a cohort of CRC long-term survivors; second, to identify sociodemographic and clinical correlates of HRQOL in these CRC survivors; third, to investigate the association of HRQOL with all-cause mortality in these individuals.

## Methods

### Study population

Between 2004 and 2007, a total of 2733 patients with histologically-proven CRC, diagnosed between 1993 and 2005, have been identified through medical records review in collaboration with surgical departments of 23 hospitals in Northern Germany and with the regional cancer registry. These patients were enrolled in a prospective study, conducted by the biobank PopGen, as reported in more detail elsewhere [24–26]. Briefly, at the time of inclusion (baseline; 2004–2007), participants were asked to fill in a questionnaire on clinical and sociodemographic characteristics and on selected lifestyle factors (e.g. cigarette smoking, alcohol consumption). The study protocol was approved by the ethics committee of the Medical Faculty of Kiel University and written informed consent was obtained from all study participants.

A first follow-up assessment was conducted from 2009 to 2011, and 2263 participants who initially agreed to be re-contacted were asked to fill in another questionnaire about clinical and sociodemographic characteristics, as well as standardized and validated questionnaires on diet (food frequency questionnaire [27]), physical activity [28], and HRQOL [29].

Of the 2263 participants re-contacted, 354 individuals were deceased and 31 had moved with unknown addresses. From 1677 individuals who filled in the HRQOL questionnaire, we excluded individuals with incomplete HRQOL data (*n* = 147), individuals with missing information on physical activity (*n* = 169), year of diagnosis (*n* = 30), and vital status (n = 30), those with implausible length of follow-up (*n* = 4), and participants with a diagnosis of small intestine cancer instead of CRC (n = 3), leaving an analytical sample of 1294 participants.

### Health-related quality of life assessment

For the assessment of HRQOL (conducted at first follow-up), the German version of the European Organisation for Research and Treatment of Cancer Quality of Life Questionnaire Core 30 (EORTC QLQ-C30; version 3.0) [29] was used. The 30-item self-report questionnaire is a validated cancer-specific instrument for the measurement of HRQOL. The QLQ-C30 is composed of a global QOL scale and of five multi-item functional scales that assess physical, role, emotional, cognitive, and social function. Furthermore, three multi-item symptom scales evaluate pain, nausea/vomiting, and fatigue, and six single-item scales measure constipation, diarrhea, appetite loss, dyspnea, insomnia, and financial difficulties. All items are scored on a scale from 1 (not at all) to 4 (very much), except for global QOL, which is scored from 1 (very poor) to 7 (excellent). A scoring procedure was applied according to the EORTC QLQ-C30 Scoring Manual [30]. All scales were linearly transformed to standardize the raw scores to scores that range from 0 to 100. High functional scores and high global QOL scores indicate high (functional) QOL whereas high symptom scores represent more severe symptoms. A summary score was calculated from 13 scales (excluding global QOL and financial difficulties) with the symptom scales being reversed (100 - symptom scale) to obtain a uniform direction of all scales [31], as follows: *QLQ-C30 summary score = (physical functioning + role functioning + social functioning + emotional functioning + cognitive functioning + (100-fatigue) + (100-pain) + (100-nausea/vomiting) + (100-dyspnea) + (100-insomnia) + (100-appetite loss) + (100-constipation) + (100-diarrhea)) / 13*.

### Assessment of sociodemographic, clinical, and lifestyle characteristics

The self-administered questionnaires on clinical and sociodemographic characteristics assessed date of diagnosis, tumor location, neoadjuvant and adjuvant types of therapy, occurrence of other types of cancer (before and after study inclusion) or occurrence of metastases, sex, age at diagnosis, age at HRQOL assessment (first follow-up), education (≤9, 10, ≥11 years, unknown) and family status (single, married, in partnership, divorced, widowed, unknown) at first follow-up, current stoma at first follow-up, smoking status at first follow-up, and postdiagnostic body weight and height at baseline and first follow-up. BMI (kg/m^2^) was calculated with weight divided by the square of height in meters. We validated self-reported clinical data (tumor location, type of therapy, metastases) against medical records in a subset of 181 participants and observed overall good agreement (87% concordance). Information on physical activities during the past 12 months was obtained with validated questions [28]. Hours per week spent with different activities (walking, cycling, sports, gardening, housework, home repair, stair climbing) were derived from these questions. To obtain comparable intensity levels, metabolic equivalent of task (MET) values, derived from the 2000 Compendium of Physical Activity [32], were assigned to each corresponding activity [33].

### Vital status ascertainment

All-cause mortality was first determined in 2014. Participants who did not respond when they were re-contacted for an extension of their informed consent, or for whom the spouse reported the study participant’s death, vital status was attained from population registries and date of death was recorded. In 2016, vital status of all participants was updated via population registries and date of death was recorded if participants were deceased. Altogether, 175 participants had died since HRQOL assessment.

### Statistical analyses

First, participant characteristics were determined using frequencies or medians and interquartile ranges (IQR) for the overall sample and for subgroups of individuals with a HRQOL summary score below and at or above the median. Differences between these subgroups were examined with the chi-squared test for categorical variables and with the Wilcoxon ranksum test for continuous variables.

Second, medians and IQRs were calculated for the summary score, global QOL, and for each functioning and symptom scale. For symptom scales, also prevalence (defined as a symptom scale > 0) were computed.

Third, in order to determine potential correlates of the different HRQOL scores, odds ratios (ORs) and 95% confidence intervals (95% CIs), derived from multivariable-adjusted logistic regression models in cross-sectional analyses, were estimated with the respective score modeled as a binary outcome (below vs. at/above the score-specific median) and with sociodemographic (sex, age, education, family status) and clinical (tumor location, metastases, other cancer, type of therapy, current stoma) characteristics as exposures (and, thus, as potential correlates). Since the residuals of our HRQOL outcome variables, when modeled as continuous traits, were not normally distributed and, thus, violated a key assumption of linear regression analyses, we modeled HRQOL as a binary trait (using the score-specific median as the cut-off) and performed logistic regression analyses using binary HRQOL traits as outcome variables, as it has been done before in other studies [34, 24]. The models were adjusted for the following variables, except the respective exposure variable of interest: sex, age at HRQOL assessment, BMI (continuous in kg/m^2^), physical activity (continuous in MET-hours/week), tumor location (colon, rectum both, unknown), occurrence of metastases (yes, no, unknown), occurrence of other types of cancer (yes, no, unknown), type of therapy (none, chemotherapy, radiation, both, unknown), and current stoma (yes, no, unknown).

Fourth, Cox proportional hazards regression models were used to estimate hazard ratios (HR) and 95% CIs of all-cause mortality for each 10-point-increment in the summary score, in the global QOL score, and in each functioning and symptom scale. The 10-point-increment was chosen because a 10-point change in QLQ-C30 scales was found to indicate a (“subjective significant”) “moderate” change in HRQOL domains [35]. The date of HRQOL assessment was the starting point for survival follow-up of this analysis and follow-up ended with date of death or date of last vital status assessment (2016) whichever came first. However, to address left truncation of survival times in our analyses, we considered the time between diagnosis and HRQOL assessment in the Cox models by using the ‘Entry=’-option in the PROC PHREG function in SAS [36]. We conducted a Cox model adjusting for sex and age at HRQOL assessment and a multivariable-adjusted model, which was additionally adjusted for BMI, physical activity, tumor location, type of therapy, occurrence of metastases, occurrence of other cancers, current stoma, education (≤9, 10, ≥11 years, unknown), family status (single, married/in partnership, divorced, widowed, unknown), and smoking status (never, former, current, unknown). We tested the proportional hazards assumption by the Schoenfeld residual method and by including time-dependent variables in the statistical model. Because age did not meet the proportional hazards assumption, a respective time-interaction-term (age x time) was included in each Cox regression model.

Fifth, Kaplan Meier survival curves were used to display unadjusted mortality rates according to quartiles of the HRQOL summary score.

Sixth, to test for nonlinearity in the association of HRQOL with all-cause mortality, a restricted cubic spline regression was conducted. For this analysis, the summary score (including information from nearly all functioning and symptom scales) was chosen as the independent variable. The knots were located on the 5th, 35th, 65th, and 95th percentile [37] and the reference value was the median (62.4 score points) of the first quartile of the summary score. The model was adjusted for the same covariates as the multivariable-adjusted Cox regression model (mentioned above).

Seventh, stratified analyses were performed to examine the role of potential effect modifiers (sex, age, BMI, education, family status, smoking status, tumor location, therapy, metastases, and current stoma) on the association between the summary score and all-cause mortality. Furthermore, we formally tested for statistical interactions by including respective cross product terms (summary score x potential effect modifier) in the statistical model predicting all-cause mortality.

Eighth, to assess the robustness of our results we performed several sensitivity analyses. We calculated medians and IQRs for the different HRQOL scales and provided the symptom prevalence separately for individuals with vs. without a diagnosis of metastases or other cancers. Furthermore, we also conducted the Cox regression analyses, relating different HRQOL measures to mortality, separately for individuals with vs. without a diagnosis of metastases or other cancers. We also considered excluding all participants who died within 12 months of HRQOL assessment in a sensitivity analysis but there was no individual who deceased within the first 12 months of follow-up.

## Results

### Participant characteristics

The characteristics of the study participants as a total sample and according to an HRQOL summary score below or at/above the median are presented in Table 1. Of the 1294 individuals, 43% were women and the median age at diagnosis was 62 years. HRQOL was assessed on average 6 years (median) after CRC diagnosis. Nearly half of the population (46%) reported a low educational status and 77% were married or in a partnership at time of HRQOL assessment. Sixteen percent of the individuals had a diagnosis of metastases, 21% reported a diagnosis of another cancer, and half of the participants (53%) had no additional cancer therapy except for surgery. A current stoma at time of HRQOL assessment was reported by 12% of the CRC survivor cohort. Participants with a higher HRQOL had a lower BMI, were more likely to have had a tumor located in the colon, were less likely to have had a diagnosis of metastases or other cancers, were more likely to have had no additional therapy to surgery, and were less likely to have undergone chemotherapy and radiation combined (Table 1).

**Table 1:** Characteristics of the total sample of 1294 CRC long-term survivors and according to an HRQOL summary score below or at/above the median

Participant characteristics	Total sample	Summary score < median	Summary score ≥ median	p^a^
Total no. of individuals, n	1294	647	647	
No. of deaths, n (%)	175 (14)	117 (18)	58 (9)	< 0.0001
Sex, n (%)				
Men	740 (57)	362 (56)	378 (58)	
Women	554 (43)	285 (44)	269 (42)	0.37
Age at diagnosis, y	62 (56–66)	62 (56–66)	61 (57–65)	0.27
Age at HRQOL assessment, y	69 (64–73)	69 (63–74)	69 (64–73)	0.25
Time between CRC diagnosis and HRQOL assessment, y	6 (5–8)	6 (5–8)	6 (5–8)	0.37
BMI, kg/m^2^	26.2 (23.9–29.2)	26.4 (24.0–29.4)	26.0 (23.7–28.9)	0.02
Physical activity, MET-hours/week	101 (65–149)	102 (64–144)	100 (66–152)	0.35
Education, n (%)				
Low	597 (46)	311 (48)	286 (44)	
Middle	393 (30)	196 (30)	197 (30)	
High	292 (23)	135 (21)	157 (24)	
Unknown	12 (1)	5 (1)	7 (1)	0.39
Family status, n (%)				
Single	52 (4)	27 (4)	25 (4)	
Married or in a partnership	991 (77)	482 (75)	509 (79)	
Divorced	65 (5)	37 (6)	28 (4)	
Widowed	147 (11)	76 (12)	71 (11)	
Unknown	39 (3)	25 (4)	14 (2)	0.26
Smoking status, n (%)				
Never	509 (39)	238 (37)	271 (42)	
Former	649 (50)	342 (53)	307 (47)	
Current	116 (9)	56 (9)	60 (9)	
Unknown	20 (2)	11 (2)	9 (1)	0.22
Tumor location, n (%)				
Colon	613 (47)	278 (43)	335 (52)	
Rectum	552 (43)	293 (45)	259 (40)	
Both	58 (4)	39 (6)	19 (3)	
Unknown	71 (5)	37 (6)	34 (5)	0.002
Metastases, n (%)				
Yes	209 (16)	124 (19)	85 (13)	
No	872 (67)	429 (66)	443 (68)	
Unknown	213 (16)	94 (15)	119 (18)	0.005
Other Cancer, n (%)				
Yes	270 (21)	154 (24)	116 (18)	
No	997 (77)	482 (75)	515 (80)	
Unknown	27 (2)	11 (2)	16 (2)	0.03
Therapy, n (%)				
None	681 (53)	319 (49)	362 (56)	
Chemotherapy	285 (22)	135 (21)	150 (23)	
Radiation	40 (3)	21 (3)	19 (3)	
Chemotherapy and radiation	268 (21)	164 (25)	104 (16)	
Unknown	20 (2)	8 (1)	12 (2)	0.001
Current Stoma, n (%)				
Yes	151 (12)	89 (14)	62 (10)	
No	1130 (87)	551 (85)	579 (89)	
Unknown	13 (1)	7 (1)	6 (1)	0.06

Abbreviations: *BMI* Body mass index, *CRC* Colorectal cancer, *HRQOL* Health-related quality of life, *MET* Metabolic equivalent of task

Values are n (%) or median (interquartile range)

^a^Calculated with the chi-squared test for categorical variables and with the Wilcoxon ranksum test for continuous variables

### Health-related quality of life status in long-term colorectal cancer survivors

The HRQOL summary score had a median of 87.3 (IQR: 75.3–94.4) (Table 2). The global QOL scored lower with a median of 75.0 (58.3–83.3). The highest scores of the five functional scales were observed for role (100 (66.7–100)) and social (100 (66.7–100)) functioning with the highest possible score as the median. Physical, emotional, and cognitive functioning were a little bit lower but roughly at a comparable level (between 83.3 and 86.7; Table 2). Of the nine symptom scales, fatigue and insomnia revealed the highest median scores (22.2 (0–33.3) and 33.3 (0–33.3), respectively) and also the highest symptom prevalence (70 and 52%, respectively), indicating a higher burden of these symptoms in the present cohort. Each of the other symptom scales had a median of 0, indicating on average no or minor symptom burden. Nevertheless, more than one-third of the study participants reported any symptoms of pain (44%), dyspnea (38%), and diarrhea (36%), respectively (Table 2). The sensitivity analyses comparing CRC survivors without (*n* = 880; Additional file 1: Table S1) vs. with (*n* = 414; Additional file 1: Table S2) a diagnosis of metastases or other cancers revealed only minor differences in the various HRQOL measures between these two groups and compared to the overall sample (Table 2). We observed a slightly higher HRQOL and less symptoms in individuals who reported no metastases or other cancers (Additional file 1: Table S1 and Table S2).

**Table 2:** Median and IQR for the HRQOL summary score and its scales and symptom prevalence (defined as percent of individuals with any symptoms of the respective scale) among 1294 CRC long-term survivors

QLQ-C30 Scales	Median (IQR)	Symptom prevalence
Summary score	87.3 (75.3–94.4)	
Global QOL	75.0 (58.3–83.3)	
Functioning scales		
Physical functioning	86.7 (73.3–100)	
Role functioning	100 (66.7–100)	
Emotional functioning	83.3 (66.7–100)	
Cognitive functioning	83.3 (66.7–100)	
Social functioning	100 (66.7–100)	
Symptom scales		
Fatigue	22.2 (0–33.3)	70%
Nausea and vomiting	0 (0–0)	12%
Pain	0 (0–33.3)	44%
Dyspnea	0 (0–33.3)	38%
Insomnia	33.3 (0–33.3)	52%
Appetite loss	0 (0–0)	14%
Constipation	0 (0–0)	24%
Diarrhea	0 (0–33.3)	36%
Financial difficulties	0 (0–0)	23%

Abbreviations: *CRC* Colorectal cancer, *HRQOL* Health-related quality of life, *IQR* Interquartile range, *QLQ-C30* Quality of Life Questionnaire Core 30, *QOL* Quality of life

### Correlates of health-related quality of life

Relevant correlates for low values (below the score-specific median) of the different HRQOL scales are provided in Table 3. In general, older age (except for emotional and social functioning), lower education, tumor location in both the colon and the rectum, metastases or other cancers, a combination of chemotherapy and radiation therapy, and a current stoma were statistically significant correlates of low HRQOL in cross-sectional analyses (Table 3).

**Table 3:** ORs^a^ and 95% CIs for low (defined as values below the scale-specific median) HRQOL scales according to sociodemographic and clinical characteristics in CRC survivors (n = 1294) from cross-sectional analyses

Characteristics	n	Summary score < median	Global QOL < median	Physical functioning scale <median	Role functioning scale <median	Emotional functioning scale <median	Social functioning scale <median	Cognitive functioning scale <median
		OR (95% CI)^b^	OR (95% CI)^b^	OR (95% CI)^b^	OR (95% CI)^b^	OR (95% CI)^b^	OR (95% CI)^b^	OR (95% CI)^b^
Sex								
Male	740	1 (Ref.)	1 (Ref.)	1 (Ref.)	1 (Ref.)	1 (Ref.)	1 (Ref.)	1 (Ref.)
Female	554	1.19 (0.94–1.51)	0.95 (0.75–1.21)	1.65 (1.29–2.11)	0.96 (0.75–1.22)	1.01 (0.80–1.27)	0.61 (0.48–0.78)	0.78 (0.62–0.99)
Age, years								
< 60	192	1.31 (0.93–1.86)	1.21 (0.85–1.71)	0.85 (0.60–1.21)	1.21 (0.85–1.72)	1.38 (0.98–1.96)	1.46 (1.02–2.10)	1.19 (0.85–1.68)
60–69	520	1 (Ref.)	1 (Ref.)	1 (Ref.)	1 (Ref.)	1 (Ref.)	1 (Ref.)	1 (Ref.)
70–79	479	1.21 (0.94–1.57)	1.20 (0.93–1.56)	1.70 (1.31–2.22)	1.15 (0.89–1.49)	0.97 (0.75–1.25)	0.77 (0.59–1.01)	1.29 (1.00–1.66)
≥ 80	103	1.58 (1.00–2.49)	1.85 (1.15–2.96)	3.91 (2.34–6.52)	2.07 (1.30–3.29)	0.89 (0.57–1.39)	0.58 (0.36–0.94)	1.85 (1.17–2.91)
Education								
Low	597	1 (Ref.)	1 (Ref.)	1 (Ref.)	1 (Ref.)	1 (Ref.)	1 (Ref.)	1 (Ref.)
Middle	393	0.99 (0.76–1.30)	0.68 (0.52–0.89)	0.96 (0.73–1.26)	0.95 (0.73–1.25)	0.95 (0.73–1.23)	1.09 (0.82–1.43)	1.04 (0.80–1.35)
High	292	0.86 (0.64–1.16)	0.58 (0.43–0.78)	0.67 (0.49–0.91)	0.87 (0.64–1.17)	0.89 (0.66–1.19)	0.75 (0.55–1.02)	0.79 (0.59–1.06)
Family status								
Single	52	1 (Ref.)	1 (Ref.)	1 (Ref.)	1 (Ref.)	1 (Ref.)	1 (Ref.)	1 (Ref.)
Married/in partnership	991	0.96 (0.54–1.72)	0.87 (0.49–1.57)	0.73 (0.40–1.34)	0.86 (0.48–1.54)	0.98 (0.55–1.74)	1.14 (0.63–2.09)	1.16 (0.65–2.07)
Divorced	65	1.35 (0.63–2.89)	0.98 (0.46–2.09)	0.99 (0.45–2.18)	0.97 (0.45–1.08)	0.97 (0.46–2.05)	1.09 (0.49–2.41)	1.21 (0.57–2.55)
Widowed	147	0.97 (0.50–1.89)	1.14 (0.58–2.24)	0.67 (0.33–1.37)	0.82 (0.42–1.60)	0.94 (0.49–1.82)	1.02 (0.51–2.06)	1.38 (0.71–2.67)
Tumor location								
Colon	613	1 (Ref.)	1 (Ref.)	1 (Ref.)	1 (Ref.)	1 (Ref.)	1 (Ref.)	1 (Ref.)
Rectum	552	1.11 (0.84–1.45)	1.02 (0.77–1.33)	1.33 (1.01–1.76)	1.28 (0.98–1.69)	1.03 (0.79–1.35)	1.41 (1.07–1.86)	0.90 (0.68–1.17)
Both	58	1.95 (1.08–3.54)	1.48 (0.82–2.68)	1.39 (0.77–2.53)	1.23 (0.69–2.19)	0.83 (0.47–1.45)	1.19 (0.66–2.16)	1.14 (0.65–2.02)
Metastases								
No	872	1 (Ref.)	1 (Ref.)	1 (Ref.)	1 (Ref.)	1 (Ref.)	1 (Ref.)	1 (Ref.)
Yes	209	1.43 (1.02–2.01)	1.11 (0.79–1.55)	1.29 (0.91–1.83)	1.04 (0.74–1.46)	1.14 (0.81–1.59)	1.47 (1.03–2.10)	1.33 (0.95–1.86)
Other Cancer								
No	997	1 (Ref.)	1 (Ref.)	1 (Ref.)	1 (Ref.)	1 (Ref.)	1 (Ref.)	1 (Ref.)
Yes	270	1.39 (1.05–1.85)	1.38 (1.03–1.83)	1.32 (0.99–1.77)	1.38 (1.04–1.84)	1.03 (0.78–1.37)	1.60 (1.20–2.15)	1.35 (1.02–1.79)
Therapy								
None	681	1 (Ref.)	1 (Ref.)	1 (Ref.)	1 (Ref.)	1 (Ref.)	1 (Ref.)	1 (Ref.)
Chemotherapy	285	0.90 (0.66–1.23)	1.03 (0.75–1.40)	1.13 (0.82–1.56)	1.07 (0.78–1.47)	0.90 (0.66–1.23)	1.21 (0.88–1.68)	0.93 (0.68–1.27)
Radiation	40	1.02 (0.52–1.99)	1.59 (0.79–3.20)	0.89 (0.44–1.82)	0.89 (0.45–1.76)	0.66 (0.34–1.29)	1.74 (0.87–3.50)	0.75 (0.38–1.46)
Chemotherapy + Radiation	268	1.56 (1.11–2.18)	1.57 (1.12–2.20)	0.96 (0.68–1.35)	1.62 (1.16–2.27)	1.04 (0.74–1.45)	2.33 (1.64–3.31)	1.33 (0.96–1.85)
Current stoma								
No	1130	1 (Ref.)	1 (Ref.)	1 (Ref.)	1 (Ref.)	1 (Ref.)	1 (Ref.)	1 (Ref.)
Yes	151	1.11 (0.76–1.62)	1.30 (0.88–1.92)	2.13 (1.42–3.20)	1.87 (1.27–2.77)	1.46 (1.00–2.14)	2.44 (1.60–3.71)	1.03 (0.71–1.50)

Abbreviations: *BMI* Body mass index, *CI* Confidence interval, *CRC* Colorectal cancer, *HRQOL* Health-related quality of life, *MET* Metabolic equivalent hours of task, *OR* Odds ratio, *QOL* Quality of life, *Ref* Reference

^a^Calculated with a multivariable-adjusted logistic regression model

^b^Adjusted for sex, age at HRQOL assessment, BMI, physical activity, tumor location, metastases, other cancer, therapy, and stoma; except the exposure variable of interest

Specifically, women had a statistically significantly higher risk of low physical functioning than men but a lower risk of low social and cognitive functioning as compared with men. With respect to age, younger survivors (< 60 years) had higher odds and older survivors (≥80 years) had lower odds for low social functioning as compared to survivors aged 60–69 years. A high educational level was significantly associated with decreased risk of low global QOL and low physical functioning. Rectal tumor survivors were more likely to have a low physical and social functioning than colon tumor survivors. Individuals with a diagnosis in both locations had nearly two times the odds of a low summary score. Metastases had a negative impact on the HRQOL summary score and on social functioning whereas a history of other types of cancer affected the HRQOL summary score and global QOL, as well as role, social, and cognitive functioning. The combination of chemotherapy and radiation was associated with a low HRQOL summary score, low global QOL, and low role und social functioning. Individuals with a current stoma at time of HRQOL assessment were more likely to have a low physical, role, emotional, and social functioning, as shown in Table 3.

### Association between health-related quality of life and all-cause mortality

**Fig. 1:**
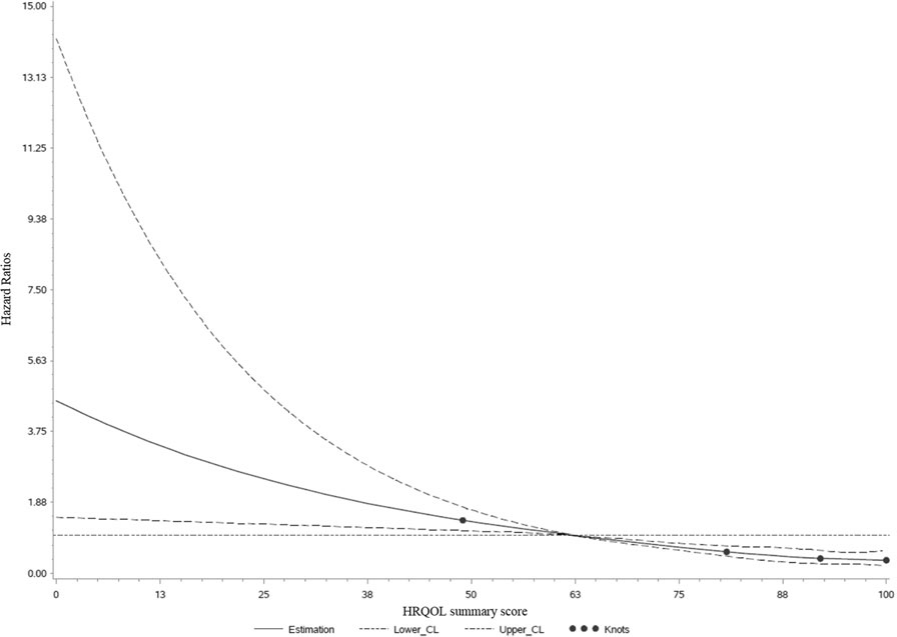
Multivariable-adjusted hazard ratios for all-cause mortality according to the HRQOL summary score in CRC survivors (*n* = 1294), calculated with restricted cubic spline regression. The solid line depicts hazard ratios and the dashed lines are the 95% CIs. The points indicate the knots at the 5th, 35th, 65th, and 95th percentiles. The reference value is the median (62.4 score points) of the first quartile of the summary score. The model was adjusted for sex, age at HRQOL assessment, BMI, physical activity, tumor location, occurrence of metastases, occurrence of other cancer, therapy, education, family status, and smoking status. The p value for overall association is < 0.0001 and the p value for nonlinearity is 0.87 (Wald chi-square test). Abbreviations: BMI, body mass index; CRC, colorectal cancer; HRQOL, health-related quality of life

After a median follow-up time of 7 years after HRQOL assessment, 175 (13.5%) of the 1294 participants had died. In the group of individuals with a lower HRQOL (<median of summary score; *n* = 647) 117 deaths (18%) were observed. Of the CRC survivors with a summary score ≥ median (n = 647) 58 individuals (9%) had died during follow-up. The Kaplan-Meier curves revealed differences in survival curves according to HRQOL quartiles with better survival in individuals with a higher HRQOL summary score (log rank test, *p* < 0.0001; Additional file 1: Figure S1). Additionally, higher scores of the HRQOL summary score and of the global QOL score were associated with improved survival (HR: 0.76; 95% CI: 0.70–0.82 and HR: 0.80; 95% CI: 0.75–0.86 for all-cause mortality per 10-point-increment, respectively) in the Cox proportional hazard regression analyses (Table 4). Restricted cubic spline regression revealed a linear association between the HRQOL summary score and all-cause mortality (p < 0.0001 for overall association; *p* = 0.87 for nonlinearity; Fig. 1).

**Table 4:** HRs^a^ and 95% CIs of all-cause mortality per 10-point-increments of QLQ-C30 scales in CRC survivors (*n* = 1294)

	Age- & sex-adjusted HR (95% CI)	Multivariable-adjusted^b^ HR (95% CI)
Summary score^c^	0.76 (0.70–0.82)	0.76 (0.70–0.82)
Global QOL^c^	0.80 (0.75–0.85)	0.80 (0.75–0.86)
Functioning Scales^c^		
Physical Functioning	0.78 (0.74–0.83)	0.80 (0.75–0.86)
Role Functioning	0.86 (0.82–0.90)	0.87 (0.83–0.91)
Emotional Functioning	0.89 (0.84–0.94)	0.88 (0.83–0.94)
Social Functioning	0.86 (0.82–0.90)	0.87 (0.83–0.91)
Cognitive Functioning	0.94 (0.88–1.01)	0.95 (0.88–1.02)
Symptom Scales^d^		
Pain	1.11 (1.06–1.16)	1.10 (1.05–1.16)
Nausea/Vomiting	1.31 (1.21–1.43)	1.31 (1.19–1.43)
Fatigue	1.21 (1.14–1.27)	1.19 (1.13–1.26)
Insomnia	1.08 (1.04–1.13)	1.08 (1.03–1.13)
Dyspnea	1.14 (1.09–1.19)	1.13 (1.08–1.19)
Appetite Loss	1.19 (1.12–1.27)	1.18 (1.11–1.26)
Constipation	1.08 (1.02–1.14)	1.09 (1.03–1.15)
Diarrhea	1.02 (0.97–1.08)	1.03 (0.97–1.09)
Financial Difficulties	1.09 (1.03–1.15)	1.07 (1.01–1.14)

Abbreviations: *BMI* Body mass index, *CI* Confidence interval, *CRC* Colorectal cancer, *HR* Hazard ratio, *QLQ-C30* Quality of life questionnaire core 30, *QOL* Quality of life

Values were calculated for a 10-point-increment in scales

^a^Calculated with Cox proportional hazards regression model

^b^Adjusted for sex, age at HRQOL assessment, BMI, physical activity, tumor location, type of therapy, metastases, other cancer, current stoma, education, family status, smoking status, and (age x time)

^c^Higher scores of the summary score, the global QOL, and the functioning scales indicate a higher HRQOL or a higher functioning

^d^Higher scores of the symptom scales indicate a higher extent of symptoms

Furthermore, every functioning scale was statistically significantly inversely related to all-cause mortality, except for cognitive functioning which was borderline non-significant (HR: 0.95; 95% CI: 0.88–1.02), with physical functioning displaying the strongest association (HR: 0.80; 95% CI: 0.75–0.86; Table 4).

Each of the symptom scales, except for diarrhea (HR: 1.03; 95% CI: 0.97–1.09), was statistically significantly positively associated with all-cause mortality, with financial difficulties displaying the weakest (HR: 1.07; 95% CI: 1.01–1.14) and nausea and vomiting (HR: 1.31; 95% CI: 1.19–1.43), fatigue (HR: 1.19; 95% CI: 1.13–1.26), and appetite loss (HR: 1.18; 95% CI: 1.11–1.26) displaying the strongest associations after multivariable adjustment (Table 4).

The stratification by potential effect modifiers revealed a stronger association between the HRQOL summary score and survival for individuals who had no therapy in addition to surgery as compared to individuals who had either chemotherapy or radiation or both chemotherapy and radiation (p_interaction_ = 0.02). Furthermore, participants with a high educational status showed a stronger association between HRQOL and all-cause mortality than participants with a low or middle educational status (p_interaction_ = 0.03). The association was also stronger in individuals with a current stoma than in those without a stoma although the interaction term was not statistically significant (p_interaction_ = 0.08; Table 5).

**Table 5:** HRs^a^ and 95% CIs of all-cause mortality for a 10-point-increment in HRQOL summary score among CRC survivors (*n* = 1294); stratified by potential effect modifiers

Potential effect modifiers	Total no. of individuals	No. of deaths	Age- & sex-adjusted HR (95% CI)	Multivariable-adjusted^b^ HR (95% CI)	p_interaction_^c^
Sex					
Men	740	126	0.75 (0.68–0.82)	0.74 (0.66–0.82)	
Women	554	49	0.78 (0.68–0.89)	0.75 (0.64–0.86)	0.48
Age at HRQOL assessment, years^d^					
< 69	626	50	0.78 (0.68–0.91)	0.88 (0.74–1.03)	
≥ 69	668	125	0.74 (0.68–0.80)	0.72 (0.66–0.80)	0.81
BMI, kg/m^2^					
< 25	497	72	0.68 (0.61–0.77)	0.67 (0.58–0.77)	
25- < 30	558	75	0.78 (0.69–0.89)	0.82 (0.72–0.94)	
≥ 30	239	28	0.81 (0.68–0.97)	0.76 (0.60–0.97)	0.19
Education					
Low	597	96	0.79 (0.72–0.88)	0.80 (0.72–0.89)	
Middle	393	44	0.81 (0.68–0.96)	0.79 (0.65–0.97)	
High	292	34	0.58 (0.48–0.69)	0.57 (0.46–0.71)	0.03
Family status					
Married/in partnership	991	127	0.77 (0.70–0.84)	0.77 (0.70–0.86)	
Single, divorced or widowed	264	43	0.75 (0.64–0.88)	0.75 (0.64–0.89)	0.84
Smoking status					
Never	509	53	0.81 (0.70–0.94)	0.81 (0.68–0.95)	
Former	649	105	0.74 (0.67–0.82)	0.72 (0.65–0.81)	
Current	116	14	0.78 (0.59–1.03)	0.74 (0.49–1.13)	0.22
Tumor location					
Colon	613	72	0.77 (0.68–0.87)	0.78 (0.68–0.88)	
Rectum	552	84	0.77 (0.69–0.86)	0.76 (0.67–0.87)	0.19
Therapy					
None	681	95	0.68 (0.61–0.76)	0.67 (0.60–0.76)	
Chemotherapy or radiation	325	47	0.87 (0.75–1.01)	0.88 (0.73–1.04)	
Both	268	31	0.78 (0.65–0.95)	0.87 (0.70–1.08)	0.02
Metastases					
Yes	209	50	0.76 (0.65–0.89)	0.78 (0.66–0.92)	
No	872	95	0.76 (0.69–0.85)	0.75 (0.67–0.85)	0.71
Current stoma					
Yes	151	30	0.69 (0.57–0.84)	0.60 (0.46–0.79)	
No	1130	145	0.78 (0.72–0.85)	0.79 (0.72–0.86)	0.08

Abbreviations: *BMI* Body mass index, *CI* Confidence interval, *CRC* Colorectal cancer, *HR* Hazard ratio, *HRQOL* Health-related quality of life

^a^Calculated with Cox proportional hazards regression model

^b^Adjusted for sex, age at HRQOL assessment, BMI, physical activity, tumor location, therapy, metastases, other cancer, current stoma, education, family status, smoking status, and (age x time); except the stratifying variable

^c^Calculated by including the cross product of the summary score and the respective potential effect modifier in the Cox proportional hazards regression model

^d^Cut-point based on median value

In sensitivity analysis, stratifying our sample by CRC survivors with (*n* = 414) and without (*n* = 880) a diagnosis of metastases or other cancers, the association between the different HRQOL scales and all-cause mortality was similar in both subgroups (Additional file 1: Table S3 and Table S4) and largely unchanged as compared to the overall sample (Table 4). Effect sizes and directions as well as measures of significance were similar, except for insomnia and financial difficulties that lost their statistically significant association with mortality in the group of CRC survivors who had a diagnosis of metastases or other cancers.

## Discussion

In the present analyses, we describe in detail the HRQOL in long-term survivors of CRC, assess cross-sectional correlates of this HRQOL (and its different scales), and evaluate the prospective association of HRQOL with all-cause mortality in these CRC survivors. Our main observations were as follows: First, in general, the overall HRQOL, obtained approximately 6 years after the cancer diagnosis, seems to be relatively high. Role and social functioning reached the highest median scores out of the five functioning scales, while out of the nine symptom scales, fatigue and insomnia had the highest median scores, indicating the highest extent of these symptoms as compared to the other symptoms. Second, sex, age, education, tumor location, metastases, other cancers, type of therapy, and current stoma were statistically significant correlates of different HRQOL scales. Third, the summary score and the global QOL as well as nearly all functioning and symptom scales were statistically significantly associated with all-cause mortality in the sense that higher HRQOL and better functioning were associated with better overall survival and more symptoms were related to worse overall survival. Fourth, the inverse association between the HRQOL summary score and all-cause mortality was stronger in individuals who had no neoadjuvant or adjuvant therapy as compared to individuals with chemotherapy or both chemotherapy and radiation and stronger in individuals with a high educational status than in individuals with a low or middle educational status.

### Health-related quality of life status

Compared to our study, previous studies reported similar high HRQOL values in CRC survivors, which are considered to be an indication of overall good QOL [1, 15, 38, 39]. However, the HRQOL of our CRC survivor cohort is in several aspects (especially regarding emotional, cognitive, and physical functioning) still slightly lower when compared to European general (healthy) population samples, though the HRQOL values of elderly general population groups (age categories > 60 years) approximate those of our CRC survivors [40]. Thus, it is conceivable that, on average, CRC survivors in the long term are able to gain HRQOL levels comparable to individuals from the general population with about the same age.

### Association of sociodemographic characteristics with health-related quality of life in cross-sectional analyses

In our study, women had a higher risk of a low physical functioning than men but a lower risk of a low social and cognitive functioning as compared with men. In contrast to our observations, however, a recent US study including 593 CRC survivors reported no significant difference between men and women in physical HRQOL and female gender was associated with increased risk of a low mental HRQOL [8].

Similar to our findings, the above mentioned US study reported a tendency towards a lower physical HRQOL and higher mental HRQOL in the elderly as compared to younger individuals, even though the association between age and HRQOL lost statistical significance after multivariable adjustment [8]. However, in a study of the Seattle Colorectal Cancer Family Registry, the association between older age and a higher risk of a very low physical component summary score remained statistically significant even after multivariable adjustment [22]. One possible explanation for the association of older age with low physical functioning is the higher prevalence of frailty and multiple comorbidities in the elderly [41] which might lead to worse physical functioning and decreased overall HRQOL. Similarly, lower cognitive functioning might, as well, rather be a consequence of advanced age than of cancer history [42].

In our study, a higher educational level was associated with higher global QOL and higher physical functioning which is in accordance with the above mentioned study on 593 long-term CRC survivors [8]. However, we did not assess income level which is likely to be highly correlated with educational level and which was associated with physical, social, and emotional well-being in other studies [10, 43].

With respect to the association between family status and HRQOL, the published literature is partially conflicting. Whereas in our cohort, family status displayed no evidence for an association with HRQOL, other studies reported being single, divorced or widowed or being married or in partnership to be inconsistently associated with low or high HRQOL [8, 44].

### Association of clinical characteristics with health-related quality of life in cross-sectional analyses

Regarding tumor location, other studies found either no significant association with HRQOL [22] or a lower HRQOL for rectal cancer survivors than for colon cancer survivors [21]. In agreement with the latter study, we observed lower HRQOL values in rectal as compared to colon cancer survivors. This association might in part be explained by a higher proportion of ostomies in rectal as compared to colon cancer survivors, but also by differences in symptoms, treatment modalities, and therapy duration between colon and rectum cancer affecting HRQOL [45]. Of note, the difference in HRQOL between rectal cancer survivors and colon cancer survivors persisted in our multivariable-adjusted analyses, including adjustment for current stoma.

Comparable to our results, a French study of 207 rectal cancer survivors reported worse role and social functioning and lower global QOL scales of the EORTC QLQ-C30 in patients who received both chemotherapy and radiation as compared to patients receiving only radiation [46]. Additionally, chemotherapy or radiation alone compared to none was not associated with HRQOL in our cohort which is in line with findings from a Dutch investigation in the PROFILES registry [47]. A combined therapy of radiation and chemotherapy is likely to be indicative of a worse disease status and it might be associated with more treatment side-effects which would explain the decreased HRQOL [48].

Several other studies demonstrated that CRC survivors with a stoma had a decreased HRQOL, even in the long-term period of two to more than five years postdiagnosis [21, 49, 50]. In our analyses, one of the strongest negatively influenced HRQOL component by the presence of a stoma was the social functioning, as similar reported by a systematic review including 10 studies [20]. Stoma patients often are affected by fear, worry, dissatisfaction, and embarrassment especially when dealing with it in public areas and social relations [51].

Of note, the ability to compare results across studies has been limited by the huge variety of applied HRQOL assessment instruments (e.g. EORTC-QLQ C30, FACT-C, SF36, SF12). Overall, our observations suggest that a more severe disease stage (e.g. tumor located on both sides, diagnosis of metastases and other cancers, chemotherapy and radiation, current stoma) is associated with lower HRQOL.

### Prospective association of health-related quality of life with all-cause mortality

In our sample, a higher HRQOL was associated with a lower risk of dying which is in line with prior studies, although these studies mainly assessed HRQOL in patients with advanced disease stages [52]. Consistently, in our study, higher values in the different functioning scales and lower values in the symptom scales were associated with longer survival. In agreement with these observations, a very low physical component score (<10th percentile) was associated with a higher risk of mortality in 1021 long-term CRC survivors of the Seattle Colorectal Cancer Family Registry (HRQOL approximately 5.5 years postdiagnosis; HR: 3.97; 95% CI: 2.95–5.34) [22].

A few studies used the same HRQOL assessment instrument as we did (EORTC QLQ-C30) and reported likewise significant associations with survival, but those studies assessed HRQOL of CRC patients very shortly after diagnosis and therapy (≤1 year) or even prior to cancer treatment [11, 53–57]; and some of these studies focused on advanced CRC [55, 56]. We expand those results by examining HRQOL in a relatively large sample (*n* = 1294) of long-term CRC survivors.

The underlying mechanisms of the association between HRQOL and survival in cancer patients are not yet entirely clear. It is conceivable that individuals with a worse HRQOL have more severe CRC or more comorbid conditions. We adjusted our analyses for the prevalence of metastases and other cancers as well as for type of therapy, but we could not control for tumor stage, recurrence, and comorbidities because of lack of information regarding these clinical characteristics. Another potential explanation for the observed association between HRQOL and survival might be psychological distress. It has been reported that individuals with psychological distress rate their HRQOL lower and that psychological distress is associated with increased cancer mortality [58, 59] and increased all-cause mortality in the general population [60]. Psychological stress and depression might adversely affect cardiovascular physiology [61] and could lead to increased inflammatory responses and cortisol release by dysregulating the hypothalamic-pituitary-adrenal axis [62].

Out of the five functioning scales, we observed the strongest association with all-cause mortality for physical functioning (HR: 0.80; 95% CI: 0.75–0.86) which might be due to the fact that physical functioning is the most affected by bodily health and fitness which is related to morbidity and mortality [63]. The strong associations between nausea/vomiting and appetite loss and survival could be due to malnutrition, cachexia, or weight loss leading to increased morbidity and mortality [64–66]. Furthermore, fatigue which was also significantly associated with mortality in our cohort has been shown to be associated with mortality even in the general population [67].

### Strengths and limitations

Strengths of our study include the large sample size, the prospective design regarding survival analyses with a long follow-up period (median, 7 years), and the validated ascertainment of vital status. Furthermore, HRQOL was assessed with one of the most widely used cancer-specific instruments (EORTC QLQ-C30).

However, there are some limitations that should be noted. Our analyses on correlates of HRQOL were cross-sectional, precluding causal inferences. Furthermore, we did not have information on comorbidities although it is likely that HRQOL as well as survival are affected by certain comorbidities. Additionally, we only had information available on all-cause mortality, but not on disease-specific mortality. Also, information on tumor stage was not available in our cohort. We only had information on metastases and other cancers. Interestingly, a recent review reported inconclusive results regarding the association between tumor stage and HRQOL [68]. Furthermore, HRQOL was assessed only once in our cohort, so that we were not able to analyze changes of HRQOL over time. In addition, HRQOL was not assessed after a fixed period of time after cancer diagnosis, but time period from cancer diagnosis until HRQOL assessment varied slightly between participants. To account for these differences and left truncation, we included a variable of time from CRC diagnosis until HRQOL assessment in our survival analyses. The data on clinical and lifestyle factors were based on self-report, which is why we cannot completely exclude the possibility of recall bias. However, the validation of self-reported clinical data against medical records in a subset of 181 patients revealed a concordance of about 87%. Of note, our sample had a relatively narrow age range, with a median age of 69 years (IQR: 64–73 years). The generalizability of our observations to other age groups of CRC survivors is unknown. Furthermore, because we assessed HRQOL at a median of 6 years after cancer diagnosis and started our survival analyses at this time point, disregarding individuals who had died before exposure (HRQOL) assessment, survivorship bias might be present in this study. Hence, the generalizability to all CRC patients is unclear, which is why we clearly characterize our study participants as long-term CRC survivors. However, we addressed left truncation and survival bias by using the ‘Entry=’-option with the variable ‘survival time from diagnosis until HRQOL assessment’ in our Cox models in the SAS program.

## Conclusions

The HRQOL in CRC survivors seems to be relatively high in the long term. Sex, age, education, tumor location, metastases, other cancers, type of therapy, and current stoma were associated with overall HRQOL (summary score and global QOL) and with different HRQOL scales. Furthermore, lower HRQOL was associated with increased all-cause mortality among CRC long-term survivors. Therefore, it is important to monitor HRQOL in long-term CRC survivors, particularly since various intervention programs, like physical activity interventions, educational programs, and psychotherapeutic interventions, might be helpful to further improve HRQOL [10]. Identifying risk factors for HRQOL deterioration may enable a better individualized care of CRC survivors. Thus, randomized controlled trials are needed to bring light into the causal relationship of clinical and sociodemographic, as well as lifestyle, determinants with HRQOL. Special support may be needed for individuals who have multiple risk factors for poor HRQOL.

## Additional file

Additional file 1: **Table S1**. Sensitivity analysis: Median and IQR for the HRQOL summary score and its scales and symptom prevalence (defined as percent of individuals with any symptoms of the respective scale) among 880 CRC long-term survivors (excluding 414 CRC survivors with a diagnosis of metastases or other cancers); **Table S2** Sensitivity analysis: Median and IQR for the HRQOL summary score and its scales and symptom prevalence (defined as percent of individuals with any symptoms of the respective scale) among 414 CRC long-term survivors with a diagnosis of metastases or other cancers; **Figure S1** Kaplan-Meier-Curves of overall survival of 1294 CRC survivors according to quartiles of the HRQOL summary score. The log-rank *p* value is < 0.0001. Abbreviations: CRC, colorectal cancer.; **Table S3** Sensitivity Analysis: HRs^a^ and 95% CIs of all-cause mortality per 10-point-increments of QLQ-C30 scales in CRC survivors (*n* = 880) after excluding individuals with a diagnosis of metastases or other cancers (*n* = 414); **Table S4** Sensitivity Analysis: HRs^a^ and 95% CIs of all-cause mortality per 10-point-increments of QLQ-C30 scales in CRC survivors with a diagnosis of metastases or other cancers (n = 414) (DOCX 41 kb)

## Abbreviations

BMI: Body mass index
CRC: Colorectal cancer
EORTC QLQ-C30: European Organisation for Research and Treatment of Cancer Quality of Life Questionnaire Core 30
HRQOL: Health-related quality of life
MET: Metabolic equivalent of task
QOL: Quality of life
