# Supplementary material for: Health-related quality of life in long-term survivors of colorectal cancer and its association with all-cause mortality: a German cohort study
Source: BMC Cancer. 2018 Nov 22;18:1156. doi: 10.1186/s12885-018-5075-1 (PMC6251222; doi:10.1186/s12885-018-5075-1)
Supplement: Supplementary file 1 — Table S1. Sensitivity analysis: Median and IQR for the HRQOL summary score and its scales and symptom prevalence (defined as percent of individuals with any symptoms of the respective scale) among 880 CRC long-term survivors (excluding 414 CRC survivors with a diagnosis of metastases or other cancers); Table S2 Sensitivity analysis: Median and IQR for the HRQOL summary score and its scales and symptom prevalence (defined as percent of individuals with any symptoms of the respective scale) among 414 CRC long-term survivors with a diagnosis of metastases or other cancers; Figure S1 Kaplan-Meier-Curves of overall survival of 1294 CRC survivors according to quartiles of the HRQOL summary score. The log-rank p value is < 0.0001. Abbreviations: CRC, colorectal cancer.; Table S3 Sensitivity Analysis: HRsa and 95% CIs of all-cause mortality per 10-point-increments of QLQ-C30 scales in CRC survivors (n = 880) after excluding individuals with a diagnosis of metastases or other cancers (n = 414); Table S4 Sensitivity Analysis: HRsa and 95% CIs of all-cause mortality per 10-point-increments of QLQ-C30 scales in CRC survivors with a diagnosis of metastases or other cancers (n = 414) (DOCX 41 kb) [file 12885_2018_5075_MOESM1_ESM.docx]

Additional file 1

**Health-related quality of life in long-term survivors of colorectal cancer**

**and its association with all-cause mortality: a German cohort study**

**Authors:** Ilka Ratjen, Clemens Schafmayer, Janna Enderle, Romina di Giuseppe, Sabina Waniek, Manja Koch, Greta Burmeister, Ute Nöthlings, Jochen Hampe, Sabrina Schlesinger, Wolfgang Lieb

**Address for correspondence:** Ilka Ratjen, MSc, Institute of Epidemiology, University of Kiel, University Hospital Schleswig-Holstein, Niemannsweg 11 (Haus 1), 24105 Kiel (Germany), Tel.: +49 431 500-30221, Fax: +49 431 500-30204, Email: ilka.ratjen@epi.uni-kiel.de.

**Table S1** Sensitivity analysis: Median and IQR for the HRQOL summary score and its scales and symptom prevalence (defined as percent of individuals with any symptoms of the respective scale) among 880 CRC long-term survivors (excluding 414 CRC survivors with a diagnosis of metastases or other cancers)

| **QLQ-C30 Scales** | **Median (IQR)** | **Symptom prevalence** |
| --- | --- | --- |
| **Summary score** | 88.2 (76.1 - 95.0) |  |
| **Global QOL** | 75.0 (58.3 - 83.3) |  |
| **Functioning scales** |  |  |
| Physical functioning | 93.3 (80.0 – 100) |  |
| Role functioning | 100 (66.7 – 100) |  |
| Emotional functioning | 83.3 (66.7 – 100) |  |
| Cognitive functioning | 100 (83.3 – 100) |  |
| Social functioning | 100 (66.7 – 100) |  |
| **Symptom scales** |  |  |
| Fatigue | 22.2 (0 - 33.3) | 68 % |
| Nausea and vomiting | 0 (0 – 0) | 10 % |
| Pain | 0 (0 - 33.3) | 43 % |
| Dyspnea | 0 (0 - 33.3) | 36 % |
| Insomnia | 33.3 (0 - 33.3) | 52 % |
| Appetite loss | 0 (0 – 0) | 13 % |
| Constipation | 0 (0 – 0) | 23 % |
| Diarrhea | 0 (0 - 33.3) | 34 % |
| Financial difficulties | 0 (0 – 0) | 23 % |

Abbreviations: CRC, colorectal cancer; HRQOL, health-related quality of life; IQR, interquartile range; QLQ-C30, Quality of Life Questionnaire Core 30; QOL, quality of life.

**Table S2** Sensitivity analysis: Median and IQR for the HRQOL summary score and its scales and symptom prevalence (defined as percent of individuals with any symptoms of the respective scale) among 414 CRC long-term survivors with a diagnosis of metastases or other cancers

| **QLQ-C30 Scales** | **Median (IQR)** | **Symptom prevalence** |
| --- | --- | --- |
| **Summary score** | 85.4 (73.2 - 92.7) |  |
| **Global QOL** | 75.0 (58.3 - 83.3) |  |
| **Functioning scales** |  |  |
| Physical functioning | 86.7 (73.3 – 100) |  |
| Role functioning | 83.3 (66.7 – 100) |  |
| Emotional functioning | 83.3 (66.7 – 100) |  |
| Cognitive functioning | 83.3 (66.7 – 100) |  |
| Social functioning | 83.3 (66.7 – 100) |  |
| **Symptom scales** |  |  |
| Fatigue | 22.2 (0 - 44.4) | 75 % |
| Nausea and vomiting | 0 (0 – 0) | 15 % |
| Pain | 0 (0 - 33.3) | 47 % |
| Dyspnea | 0 (0 - 33.3) | 44 % |
| Insomnia | 33.3 (0 - 33.3) | 53 % |
| Appetite loss | 0 (0 – 0) | 16 % |
| Constipation | 0 (0 – 33.3) | 27 % |
| Diarrhea | 0 (0 - 33.3) | 40 % |
| Financial difficulties | 0 (0 – 33.3) | 25 % |

Abbreviations: CRC, colorectal cancer; HRQOL, health-related quality of life; IQR, interquartile range; QLQ-C30, Quality of Life Questionnaire Core 30; QOL, quality of life.

**Figure S1** Kaplan-Meier-Curves of overall survival of 1294 CRC survivors according to quartiles of the HRQOL summary score. The log-rank p value is <0.0001. Abbreviations: CRC, colorectal cancer.

**Table S3** Sensitivity Analysis: HRs^a^ and 95% CIs of all-cause mortality per 10-point-increments of QLQ-C30 scales in CRC survivors (n=880) after excluding individuals with a diagnosis of metastases or other cancers (n=414)

|  | **Age- & sex-adjusted HR (95% CI)** | **Multivariable-adjusted^b^ HR (95% CI)** |
| --- | --- | --- |
| Summary score^c^ | 0.78 (0.70-0.87) | 0.79 (0.70-0.88) |
| Global QOL^c^ | 0.81 (0.74-0.88) | 0.83 (0.75-0.91) |
| ***Functioning Scales^c^*** |  |  |
| Physical Functioning | 0.75 (0.69-0.82) | 0.77 (0.70-0.85) |
| Role Functioning | 0.85 (0.80-0.90) | 0.86 (0.80-0.92) |
| Emotional Functioning | 0.90 (0.83-0.97) | 0.91 (0.84-0.99) |
| Social Functioning | 0.86 (0.81-0.91) | 0.88 (0.82-0.94) |
| Cognitive Functioning | 0.95 (0.87-1.04) | 0.95 (0.87-1.05) |
| ***Symptom Scales^d^*** |  |  |
| Pain | 1.11 (1.04-1.18) | 1.10 (1.03-1.18) |
| Nausea/Vomiting | 1.20 (1.03-1.41) | 1.20 (1.02-1.41) |
| Fatigue | 1.20 (1.11-1.28) | 1.17 (1.09-1.25) |
| Insomnia | 1.10 (1.03-1.16) | 1.09 (1.02-1.15) |
| Dyspnea | 1.12 (1.06-1.19) | 1.10 (1.03-1.17) |
| Appetite Loss | 1.14 (1.04-1.24) | 1.12 (1.03-1.23) |
| Constipation | 1.01 (0.93-1.09) | 1.05 (0.97-1.14) |
| Diarrhea | 0.98 (0.90-1.05) | 1.00 (0.92-1.08) |
| Financial Difficulties | 1.11 (1.04-1.19) | 1.09 (1.00-1.18) |
|  |  |  |

Values were calculated for a 10-point-increment in scales.

^a^ Calculated with Cox proportional hazards regression model.

^b^ Adjusted for sex, age at HRQOL assessment, BMI, physical activity, tumor location, type of therapy, current stoma, education, family status, smoking status, and (age x time).

^c^ Higher scores of the summary score, the global QOL, and the functioning scales indicate a higher HRQOL or a higher functioning.

^d^ Higher scores of the symptom scales indicate a higher extent of symptoms.

Abbreviations: BMI, body mass index; CI, confidence interval; CRC, colorectal cancer; HR, hazard ratio; QLQ-C30, quality of life questionnaire core 30; QOL, quality of life.

**Table S4** Sensitivity Analysis: HRs^a^ and 95% CIs of all-cause mortality per 10-point-increments of QLQ-C30 scales in CRC survivors with a diagnosis of metastases or other cancers (n=414)

|  | **Age- & sex-adjusted HR (95% CI)** | **Multivariable-adjusted^b^ HR (95% CI)** |
| --- | --- | --- |
| Summary score^c^ | 0.75 (0.67-0.84) | 0.76 (0.67-0.87) |
| Global QOL^c^ | 0.81 (0.73-0.89) | 0.82 (0.74-0.90) |
| ***Functioning Scales^c^*** |  |  |
| Physical Functioning | 0.83 (0.76-0.91) | 0.85 (0.77-0.94) |
| Role Functioning | 0.88 (0.82-0.94) | 0.89 (0.83-0.95) |
| Emotional Functioning | 0.89 (0.82-0.98) | 0.90 (0.82-0.99) |
| Social Functioning | 0.87 (0.81-0.94) | 0.89 (0.82-0.96) |
| Cognitive Functioning | 0.94 (0.85-1.05) | 0.98 (0.88-1.09) |
| ***Symptom Scales^d^*** |  |  |
| Pain | 1.11 (1.03-1.19) | 1.11 (1.03-1.21) |
| Nausea/Vomiting | 1.37 (1.23-1.53) | 1.38 (1.22-1.57) |
| Fatigue | 1.20 (1.11-1.30) | 1.18 (1.09-1.28) |
| Insomnia | 1.06 (0.99-1.13) | 1.04 (0.97-1.12) |
| Dyspnea | 1.15 (1.08-1.23) | 1.16 (1.08-1.24) |
| Appetite Loss | 1.26 (1.15-1.39) | 1.22 (1.10-1.36) |
| Constipation | 1.16 (1.07-1.25) | 1.17 (1.07-1.27) |
| Diarrhea | 1.06 (0.99-1.14) | 1.04 (0.97-1.13) |
| Financial Difficulties | 1.06 (0.97-1.16) | 1.04 (0.95-1.15) |
|  |  |  |

Values were calculated for a 10-point-increment in scales.

^a^ Calculated with Cox proportional hazards regression model.

^b^ Adjusted for sex, age at HRQOL assessment, BMI, physical activity, tumor location, type of therapy, current stoma, education, family status, smoking status, and (age x time).

^c^ Higher scores of the summary score, the global QOL, and the functioning scales indicate a higher HRQOL or a higher functioning.

^d^ Higher scores of the symptom scales indicate a higher extent of symptoms.

Abbreviations: BMI, body mass index; CI, confidence interval; CRC, colorectal cancer; HR, hazard ratio; QLQ-C30, quality of life questionnaire core 30; QOL, quality of life.
